# (3a*R*,8b*R*)-3a,8b-Dihy­droxy-2-methyl­sulfanyl-3-nitro-1-phenyl-1,8b-di­hydro­indeno[1,2-*b*]pyrrol-4(3a*H*)-one

**DOI:** 10.1107/S1600536813029577

**Published:** 2013-11-13

**Authors:** R. A. Nagalakshmi, J. Suresh, V. Jeyachandran, R. Ranjith Kumar, P. L. Nilantha Lakshman

**Affiliations:** a Department of Physics, The Madura College, Madurai 625 011, India; b Department of Organic Chemistry, School of Chemistry, Madurai Kamaraj University, Madurai 625 021, India; c Department of Food Science and Technology, University of Ruhuna, Mapalana, Kamburupitiya 81100, Sri Lanka

## Abstract

In the title compound, C_18_H_14_N_2_O_5_, the pyrrolidine ring adopts a shallow envelope conformation, with the C atom bearing the OH group (and remote from the N atom) displaced by 0.257 (2) Å from the other atoms. The cyclo­pentane ring has a twisted conformation about the C—C bond bearing one =O and one —OH grouping. The dihedral angle between the five-membered rings (all atoms) is 65.54 (9)° and the OH groups lie to the same side of the ring-junction. The mol­ecular structure features a weak intra­molecular O—H⋯O bond and a possible C—H⋯π inter­action. In the crystal, the mol­ecules are linked into [010] chains by O—H⋯O hydrogen bonds. Weak C—H⋯O bonds connect the chains into (100) sheets.

## Related literature

For background to pyrrolidine derivatives, see: Grigg (1995 ▶); Kravchenko *et al.* (2005 ▶). For ring conformation analysis, see: Cremer & Pople (1975 ▶). For a related structure, see: Ghorbani (2012 ▶).
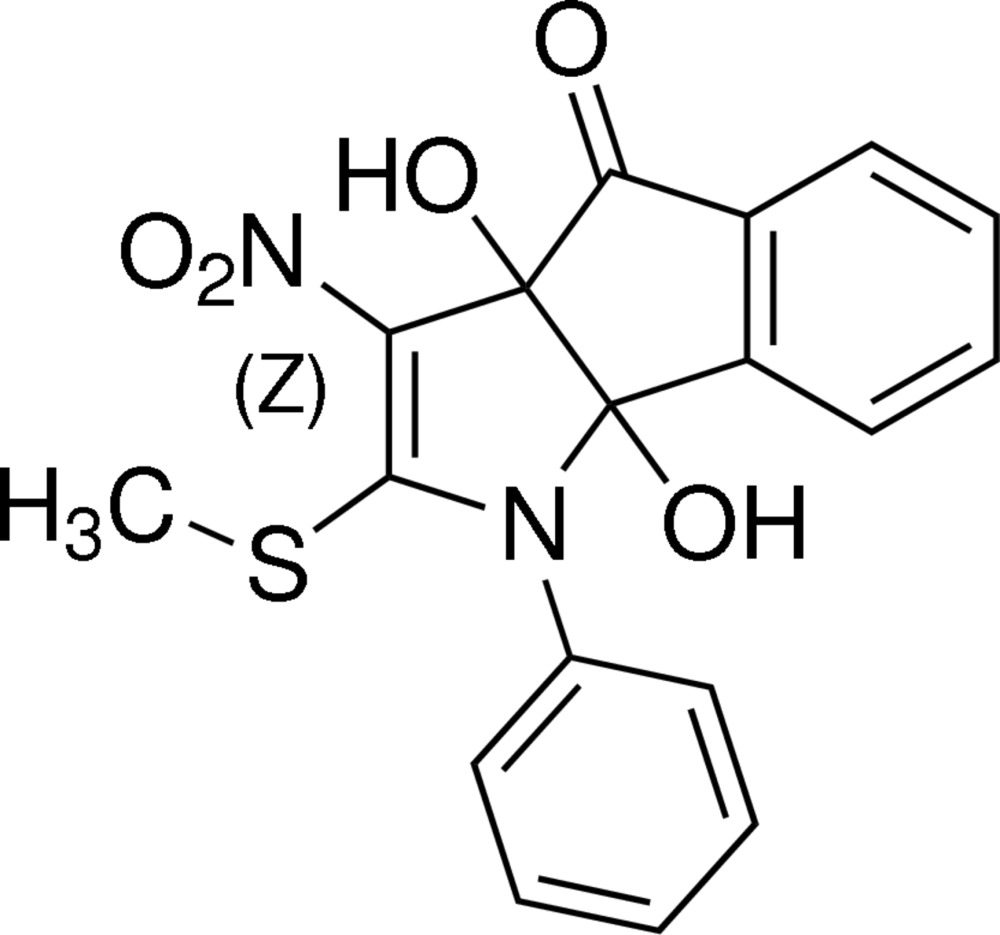


## Experimental

### 

#### Crystal data

C_18_H_14_N_2_O_5_S
*M* *_r_* = 370.37
Orthorhombic, 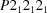

*a* = 9.6625 (3) Å
*b* = 10.8994 (2) Å
*c* = 15.8154 (4) Å
*V* = 1665.61 (7) Å^3^
*Z* = 4
Mo *K*α radiation
μ = 0.23 mm^−1^
*T* = 293 K
0.21 × 0.19 × 0.18 mm

#### Data collection

Bruker Kappa APEXII diffractometer
Absorption correction: multi-scan (*SADABS*; Sheldrick, 1996 ▶) *T*_min_ = 0.967, *T*_max_ = 0.974
9249 measured reflections
4140 independent reflections
3800 reflections with *I* > 2σ(*I*)
*R*_int_ = 0.018

#### Refinement

*R*[*F* ^2^ > 2σ(*F* ^2^)] = 0.032
*wR*(*F* ^2^) = 0.092
*S* = 1.00
4140 reflections
236 parameters
H-atom parameters constrained
Δρ_max_ = 0.23 e Å^−3^
Δρ_min_ = −0.19 e Å^−3^
Absolute structure: Flack (1983 ▶)
Absolute structure parameter: 0.00 (6)

### 

Data collection: *APEX2* (Bruker, 2004 ▶); cell refinement: *SAINT* (Bruker, 2004 ▶); data reduction: *SAINT*; program(s) used to solve structure: *SHELXS97* (Sheldrick, 2008 ▶); program(s) used to refine structure: *SHELXL97* (Sheldrick, 2008 ▶); molecular graphics: *PLATON* (Spek, 2009 ▶); software used to prepare material for publication: *SHELXL97*.

## Supplementary Material

Crystal structure: contains datablock(s) global, I. DOI: 10.1107/S1600536813029577/hb7154sup1.cif

Structure factors: contains datablock(s) I. DOI: 10.1107/S1600536813029577/hb7154Isup2.hkl

Additional supplementary materials:  crystallographic information; 3D view; checkCIF report

## Figures and Tables

**Table 1:** Hydrogen-bond geometry (Å, °) *Cg*1 is the centroid of the C13–C18 benzene ring.

*D*—H⋯*A*	*D*—H	H⋯*A*	*D*⋯*A*	*D*—H⋯*A*
O3—H3⋯O5	0.82	2.34	2.895 (2)	126
O2—H2⋯O5^i^	0.82	2.00	2.8155 (18)	171
C11—H11⋯O4^ii^	0.93	2.51	3.423 (3)	166
C9—H9⋯*Cg*1	0.93	2.89	3.545 (2)	129

Symmetry codes: (i) 
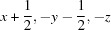
; (ii) 
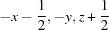
.

**Fig. 1.**
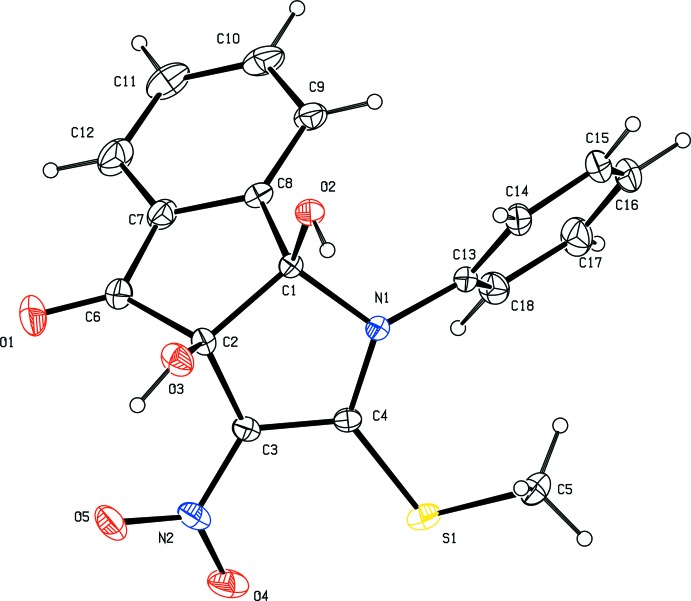
The molecular structure of (I), showing 20% probability displacement ellipsoids. H-atoms are omitted for clarity.

**Fig. 2.**
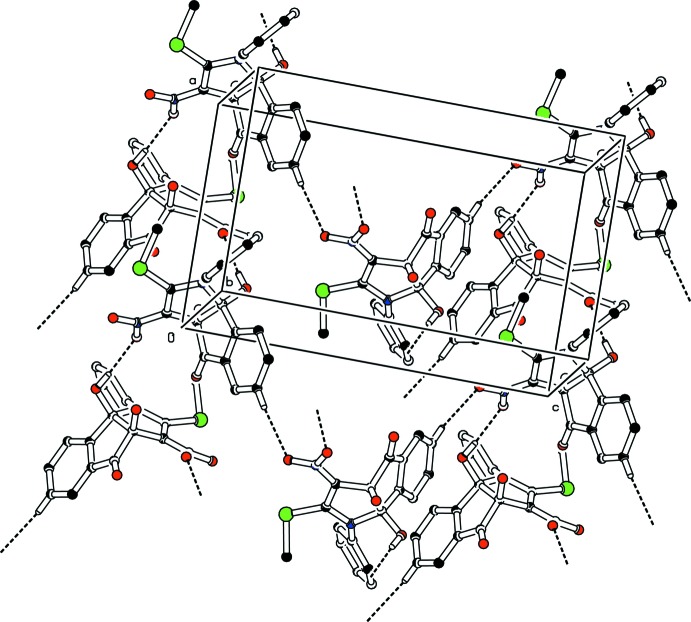
The partial packing diagram showing 0—H···O and C—H···O interactions.

## supplementary crystallographic information

### 1. Comment

Pyrrolidine-containing compounds are of significant importance because of their biological activities and widespread employment in catalysis (Grigg, 1995; Kravchenko *et al.*, 2005). As part of our own studies in this area, we have undertaken the crystal structure determination of the title compound, a pyrrolidine derivative,and the results are presented here.

In the title compound (Fig 1) C_18_H_14_N_2_O_5_, the central pyrrolidine ring is enveloped on C2 with the puckering parameters q_2_ = 0.1576 (16) Å and φ_2_ = 243.9 (6) ° (Cremer & Pople, 1975). The cyclopentane ring has a twisted conformation with the puckering parameters q_2_ = 0.1540 (18) Å and φ_2_ = 45.5 (7) °. The benzene ring of the indane ring is in planar with r.m.s. deviation 0.007 (1) Å. The benzene ring attached to the pyrrole ring is also planar with r.m.s. deviation 0.0096 Å. The aryl ring makes the dihedral angle of 57.40 (1) ° with the mean plane of the pyrrolidine ring. The sum of the C—N—C angles around N1 (359.89 (1)°) atom is implying a noticeable flattening of the trigonal pyramidal geometry about N1. The conformation of the methylsulfanyl moiety is in anticlinical conformation as evidenced from the torsion angle C5-S1-C4-C3 = -145.2 ° . The nitro group is well ordered and makes a dihedral angle of 20.25 (17) ° with the mean plane of pyrrolidine ring. The twist of the methyl sulfanyl ring attached with the pyrrolidine ring is indicated by the torsion angle C5-S1-C4-N1 = 28.5 °. The bond length C4-S1 = 1.727 (2) Å is shorter than S1-C5 = 1.804 (3) Å as found in similar structure (Ghorbani, 2012). The methyl group of the methyl sulfanyl substituent is tilted towards the plane of the benzene ring as indicated by the angle C4-S1-C5 = 106.52 (11) °. Due to conjugation ,the bond length C1-O2 = 1.380 (4)Å is shorter than the bond length C2-O3 = 1.409 (2) Å. The methoxy groups substituted at the phenyl rings are twisted, as it can be seen from the torsion angles C19-O6-C16-C17 = 14.1 (3) °.

The structure features a weak intra-molecular O—H···O interaction. An inter- molecular O2—H2···O5 inteaction forms a zig-zag chain along b axis and they are further connected by C11—H11···O4 inter-molecular interaction, forming a zig-zag chain along c axis. A weak C—H···Cg1 interaction is also observed, as in Table 1 (Cg1 is the centroid of the benzene ring C13-C18).

### 2. Experimental

A mixture of (E)-N-(1-(methylthio)-2-nitrovinyl)aniline (1 mmol) with ninhydrin (1 mmol) in presence of glacial AcOH (3-5 drops) was thoroughly ground in a pestle and mortar at room temperature for 2-10 mins. Thereaction progress was monitored by thin layer chromatography. After completion of the reaction, the reaction mixture was triturated with crushed ice, the resulting solid filtered off and washed with water to afford the pure product. The compound was further recrystallized from ethanol to obtain colourless blocks. Melting point : 451K - 453K. Yield : 94%.

### 3. Refinement

H atoms were placed at calculated positions and allowed to ride on their carrier atoms with C—H = 0.93–0.98Å. *U*_iso_ = 1.2*U*_eq_(C) for CH_2_ and CH groups and *U*_iso_ = 1.5*U*_eq_(C) for CH_3_ group.

### Crystal data

**Table:**

C_18_H_14_N_2_O_5_S	*F*(000) = 768
*M**_r_* = 370.37	*D*_x_ = 1.477 Mg m^−^^3^
Orthorhombic, *P*2_1_2_1_2_1_	Mo *K*α radiation, λ = 0.71073 Å
Hall symbol: P 2ac 2ab	Cell parameters from 2000 reflections
*a* = 9.6625 (3) Å	θ = 2–31°
*b* = 10.8994 (2) Å	µ = 0.23 mm^−^^1^
*c* = 15.8154 (4) Å	*T* = 293 K
*V* = 1665.61 (7) Å^3^	Block, colourless
*Z* = 4	0.21 × 0.19 × 0.18 mm

### Data collection

**Table:**

Bruker Kappa APEXII diffractometer	4140 independent reflections
Radiation source: fine-focus sealed tube	3800 reflections with *I* > 2σ(*I*)
Graphite monochromator	*R*_int_ = 0.018
Detector resolution: 0 pixels mm^-1^	θ_max_ = 28.3°, θ_min_ = 2.3°
ω and φ scans	*h* = −12→12
Absorption correction: multi-scan (*SADABS*; Sheldrick, 1996)	*k* = −12→14
*T*_min_ = 0.967, *T*_max_ = 0.974	*l* = −18→21
9249 measured reflections	

### Refinement

**Table:**

Refinement on *F*^2^	Secondary atom site location: difference Fourier map
Least-squares matrix: full	Hydrogen site location: inferred from neighbouring sites
*R*[*F*^2^ > 2σ(*F*^2^)] = 0.032	H-atom parameters constrained
*wR*(*F*^2^) = 0.092	*w* = 1/[σ^2^(*F*_o_^2^) + (0.0557*P*)^2^ + 0.2428*P*] where *P* = (*F*_o_^2^ + 2*F*_c_^2^)/3
*S* = 1.00	(Δ/σ)_max_ < 0.001
4140 reflections	Δρ_max_ = 0.23 e Å^−^^3^
236 parameters	Δρ_min_ = −0.19 e Å^−^^3^
0 restraints	Absolute structure: Flack (1983), 000 Friedel pairs
Primary atom site location: structure-invariant direct methods	Absolute structure parameter: 0.00 (6)

### Special details

**Table:**

Geometry. All e.s.d.'s (except the e.s.d. in the dihedral angle between two l.s. planes) are estimated using the full covariance matrix. The cell e.s.d.'s are taken into account individually in the estimation of e.s.d.'s in distances, angles and torsion angles; correlations between e.s.d.'s in cell parameters are only used when they are defined by crystal symmetry. An approximate (isotropic) treatment of cell e.s.d.'s is used for estimating e.s.d.'s involving l.s.planes.
Refinement. Refinement of *F*^2^ against ALL reflections. The weighted *R*-factor *wR* and goodness of fit *S* are based on *F*^2^, conventional *R*-factors *R* are based on *F*, with *F* set to zero for negative *F*^2^. The threshold expression of *F*^2^ > σ (*F*^2^) is used only for calculating *R*-factors(gt) *etc*. and is not relevant to the choice of reflections for refinement. *R*-factors based on *F*.^2^ are statistically about twice as large as those based on *F*, and *R*-factors based on ALL data will be even larger.

### Fractional atomic coordinates and isotropic or equivalent isotropic displacement parameters (Å^2^)

**Table:**

	*x*	*y*	*z*	*U*_iso_*/*U*_eq_	
C1	0.14622 (15)	−0.06791 (13)	0.10358 (9)	0.0281 (3)	
C2	0.07556 (17)	−0.17588 (14)	0.05420 (10)	0.0322 (3)	
C3	0.07626 (16)	−0.12630 (14)	−0.03423 (9)	0.0315 (3)	
C4	0.16436 (15)	−0.02676 (14)	−0.04222 (9)	0.0287 (3)	
C5	0.3928 (2)	0.0803 (2)	−0.11431 (14)	0.0551 (5)	
H5A	0.4342	0.1155	−0.1639	0.083*	
H5B	0.4461	0.0106	−0.0964	0.083*	
H5C	0.3910	0.1403	−0.0699	0.083*	
C6	−0.07130 (19)	−0.18225 (19)	0.09064 (12)	0.0447 (4)	
C7	−0.09638 (18)	−0.06680 (17)	0.13669 (12)	0.0426 (4)	
C8	0.02672 (16)	−0.00269 (14)	0.14699 (9)	0.0326 (3)	
C9	0.0300 (2)	0.10338 (17)	0.19568 (11)	0.0438 (4)	
H9	0.1121	0.1461	0.2044	0.053*	
C10	−0.0939 (3)	0.1434 (2)	0.23091 (13)	0.0583 (6)	
H10	−0.0941	0.2146	0.2633	0.070*	
C11	−0.2169 (3)	0.0807 (2)	0.21922 (15)	0.0663 (6)	
H11	−0.2982	0.1110	0.2428	0.080*	
C12	−0.2199 (2)	−0.0259 (2)	0.17303 (15)	0.0612 (5)	
H12	−0.3018	−0.0697	0.1661	0.073*	
C13	0.25948 (16)	0.12942 (13)	0.05639 (10)	0.0318 (3)	
C14	0.3684 (2)	0.14182 (18)	0.11299 (11)	0.0446 (4)	
H14	0.4143	0.0729	0.1335	0.054*	
C15	0.4078 (2)	0.2584 (2)	0.13854 (14)	0.0559 (5)	
H15	0.4781	0.2674	0.1782	0.067*	
C16	0.3443 (3)	0.36057 (19)	0.10607 (15)	0.0605 (6)	
H16	0.3729	0.4383	0.1229	0.073*	
C17	0.2376 (2)	0.34801 (17)	0.04819 (16)	0.0566 (5)	
H17	0.1954	0.4173	0.0255	0.068*	
C18	0.1936 (2)	0.23165 (15)	0.02401 (12)	0.0430 (4)	
H18	0.1203	0.2227	−0.0136	0.052*	
N1	0.21036 (13)	0.00884 (11)	0.03503 (7)	0.0280 (2)	
N2	0.02304 (15)	−0.19222 (14)	−0.10135 (10)	0.0405 (3)	
O1	−0.14751 (19)	−0.26911 (18)	0.08517 (13)	0.0783 (6)	
O2	0.24185 (12)	−0.10349 (10)	0.16359 (7)	0.0339 (2)	
H2	0.3065	−0.1386	0.1404	0.051*	
O3	0.14813 (16)	−0.28697 (11)	0.06474 (9)	0.0464 (3)	
H3	0.1156	−0.3392	0.0331	0.070*	
O4	0.01453 (17)	−0.14460 (16)	−0.17197 (9)	0.0568 (4)	
O5	−0.01833 (16)	−0.30050 (13)	−0.08714 (10)	0.0559 (4)	
S1	0.21819 (5)	0.03237 (4)	−0.13829 (2)	0.04134 (11)	

### Atomic displacement parameters (Å^2^)

**Table:**

	*U*^11^	*U*^22^	*U*^33^	*U*^12^	*U*^13^	*U*^23^
C1	0.0308 (7)	0.0277 (6)	0.0257 (6)	−0.0027 (5)	0.0015 (5)	0.0009 (5)
C2	0.0339 (7)	0.0293 (7)	0.0336 (7)	−0.0048 (6)	−0.0008 (6)	−0.0018 (6)
C3	0.0317 (7)	0.0334 (7)	0.0295 (7)	0.0016 (6)	−0.0019 (6)	−0.0056 (6)
C4	0.0297 (6)	0.0302 (7)	0.0262 (6)	0.0051 (6)	0.0003 (5)	−0.0009 (5)
C5	0.0551 (11)	0.0604 (12)	0.0497 (10)	−0.0134 (10)	0.0204 (9)	−0.0003 (9)
C6	0.0372 (8)	0.0548 (11)	0.0421 (9)	−0.0125 (8)	0.0044 (7)	−0.0029 (8)
C7	0.0355 (8)	0.0529 (10)	0.0395 (8)	−0.0020 (7)	0.0064 (7)	0.0023 (8)
C8	0.0355 (7)	0.0361 (7)	0.0262 (6)	0.0027 (6)	0.0047 (6)	0.0031 (6)
C9	0.0557 (11)	0.0398 (8)	0.0359 (8)	0.0028 (8)	0.0116 (8)	−0.0025 (7)
C10	0.0778 (16)	0.0532 (11)	0.0441 (10)	0.0179 (11)	0.0222 (10)	−0.0019 (9)
C11	0.0537 (13)	0.0828 (15)	0.0625 (13)	0.0234 (12)	0.0275 (11)	0.0038 (12)
C12	0.0374 (9)	0.0844 (15)	0.0616 (12)	0.0002 (11)	0.0152 (9)	0.0022 (11)
C13	0.0357 (8)	0.0300 (7)	0.0297 (7)	−0.0073 (6)	0.0046 (6)	−0.0022 (5)
C14	0.0445 (9)	0.0487 (10)	0.0408 (9)	−0.0166 (8)	−0.0042 (7)	0.0046 (7)
C15	0.0618 (12)	0.0596 (11)	0.0462 (10)	−0.0313 (10)	−0.0019 (10)	−0.0048 (10)
C16	0.0737 (14)	0.0452 (11)	0.0626 (12)	−0.0282 (10)	0.0175 (11)	−0.0168 (9)
C17	0.0615 (13)	0.0334 (8)	0.0749 (14)	−0.0024 (9)	0.0114 (11)	−0.0033 (9)
C18	0.0438 (10)	0.0353 (8)	0.0498 (10)	0.0015 (7)	0.0015 (8)	−0.0031 (7)
N1	0.0304 (6)	0.0273 (5)	0.0262 (5)	−0.0031 (5)	0.0021 (5)	−0.0003 (4)
N2	0.0346 (7)	0.0460 (8)	0.0409 (8)	0.0039 (6)	−0.0072 (6)	−0.0161 (6)
O1	0.0633 (10)	0.0836 (12)	0.0880 (12)	−0.0434 (10)	0.0184 (9)	−0.0231 (10)
O2	0.0361 (6)	0.0368 (5)	0.0288 (5)	0.0014 (5)	−0.0028 (4)	0.0014 (4)
O3	0.0615 (8)	0.0269 (5)	0.0507 (7)	0.0006 (5)	−0.0068 (6)	−0.0009 (5)
O4	0.0583 (9)	0.0755 (10)	0.0365 (7)	0.0049 (8)	−0.0143 (6)	−0.0108 (6)
O5	0.0563 (8)	0.0461 (7)	0.0652 (9)	−0.0123 (6)	−0.0069 (7)	−0.0198 (7)
S1	0.0506 (2)	0.0458 (2)	0.02760 (17)	0.00591 (19)	0.00479 (17)	0.00507 (16)

### Geometric parameters (Å, º)

**Table:**

C1—O2	1.3802 (18)	C10—C11	1.383 (4)
C1—N1	1.5030 (18)	C10—H10	0.9300
C1—C8	1.520 (2)	C11—C12	1.373 (4)
C1—C2	1.569 (2)	C11—H11	0.9300
C2—O3	1.409 (2)	C12—H12	0.9300
C2—C3	1.499 (2)	C13—C18	1.382 (2)
C2—C6	1.533 (2)	C13—C14	1.388 (2)
C3—N2	1.3811 (19)	C13—N1	1.4377 (18)
C3—C4	1.385 (2)	C14—C15	1.387 (3)
C4—N1	1.3567 (18)	C14—H14	0.9300
C4—S1	1.7304 (15)	C15—C16	1.371 (3)
C5—S1	1.807 (2)	C15—H15	0.9300
C5—H5A	0.9600	C16—C17	1.386 (4)
C5—H5B	0.9600	C16—H16	0.9300
C5—H5C	0.9600	C17—C18	1.391 (3)
C6—O1	1.202 (2)	C17—H17	0.9300
C6—C7	1.474 (3)	C18—H18	0.9300
C7—C8	1.389 (2)	N2—O4	1.234 (2)
C7—C12	1.398 (3)	N2—O5	1.266 (2)
C8—C9	1.389 (2)	O2—H2	0.8200
C9—C10	1.391 (3)	O3—H3	0.8200
C9—H9	0.9300		
			
O2—C1—N1	112.11 (12)	C11—C10—C9	122.07 (19)
O2—C1—C8	109.23 (12)	C11—C10—H10	119.0
N1—C1—C8	112.26 (11)	C9—C10—H10	119.0
O2—C1—C2	115.02 (12)	C12—C11—C10	120.5 (2)
N1—C1—C2	103.76 (11)	C12—C11—H11	119.7
C8—C1—C2	104.18 (12)	C10—C11—H11	119.7
O3—C2—C3	114.69 (13)	C11—C12—C7	118.1 (2)
O3—C2—C6	112.16 (14)	C11—C12—H12	121.0
C3—C2—C6	111.79 (14)	C7—C12—H12	121.0
O3—C2—C1	111.66 (13)	C18—C13—C14	120.66 (15)
C3—C2—C1	101.07 (12)	C18—C13—N1	119.86 (15)
C6—C2—C1	104.46 (13)	C14—C13—N1	119.39 (15)
N2—C3—C4	124.50 (14)	C15—C14—C13	119.01 (19)
N2—C3—C2	121.85 (14)	C15—C14—H14	120.5
C4—C3—C2	111.73 (13)	C13—C14—H14	120.5
N1—C4—C3	110.08 (13)	C16—C15—C14	120.83 (19)
N1—C4—S1	125.85 (11)	C16—C15—H15	119.6
C3—C4—S1	123.82 (11)	C14—C15—H15	119.6
S1—C5—H5A	109.5	C15—C16—C17	120.00 (17)
S1—C5—H5B	109.5	C15—C16—H16	120.0
H5A—C5—H5B	109.5	C17—C16—H16	120.0
S1—C5—H5C	109.5	C16—C17—C18	120.0 (2)
H5A—C5—H5C	109.5	C16—C17—H17	120.0
H5B—C5—H5C	109.5	C18—C17—H17	120.0
O1—C6—C7	127.37 (19)	C13—C18—C17	119.48 (18)
O1—C6—C2	125.13 (19)	C13—C18—H18	120.3
C7—C6—C2	107.41 (14)	C17—C18—H18	120.3
C8—C7—C12	121.52 (18)	C4—N1—C13	125.54 (12)
C8—C7—C6	110.28 (15)	C4—N1—C1	110.81 (11)
C12—C7—C6	128.00 (18)	C13—N1—C1	118.38 (11)
C7—C8—C9	120.20 (16)	O4—N2—O5	122.11 (15)
C7—C8—C1	111.24 (14)	O4—N2—C3	120.10 (15)
C9—C8—C1	128.48 (15)	O5—N2—C3	117.78 (16)
C8—C9—C10	117.6 (2)	C1—O2—H2	109.5
C8—C9—H9	121.2	C2—O3—H3	109.5
C10—C9—H9	121.2	C4—S1—C5	101.78 (8)
			
O2—C1—C2—O3	−15.37 (18)	N1—C1—C8—C9	−64.8 (2)
N1—C1—C2—O3	107.44 (14)	C2—C1—C8—C9	−176.43 (15)
C8—C1—C2—O3	−134.90 (13)	C7—C8—C9—C10	−1.7 (3)
O2—C1—C2—C3	−137.77 (13)	C1—C8—C9—C10	−178.21 (16)
N1—C1—C2—C3	−14.95 (14)	C8—C9—C10—C11	0.5 (3)
C8—C1—C2—C3	102.70 (13)	C9—C10—C11—C12	1.2 (4)
O2—C1—C2—C6	106.06 (15)	C10—C11—C12—C7	−1.7 (4)
N1—C1—C2—C6	−131.12 (13)	C8—C7—C12—C11	0.4 (3)
C8—C1—C2—C6	−13.47 (16)	C6—C7—C12—C11	174.7 (2)
O3—C2—C3—N2	59.5 (2)	C18—C13—C14—C15	1.8 (3)
C6—C2—C3—N2	−69.58 (19)	N1—C13—C14—C15	−174.80 (16)
C1—C2—C3—N2	179.80 (14)	C13—C14—C15—C16	−2.6 (3)
O3—C2—C3—C4	−105.35 (15)	C14—C15—C16—C17	1.3 (3)
C6—C2—C3—C4	125.53 (15)	C15—C16—C17—C18	0.9 (3)
C1—C2—C3—C4	14.91 (16)	C14—C13—C18—C17	0.3 (3)
N2—C3—C4—N1	−173.08 (14)	N1—C13—C18—C17	176.95 (17)
C2—C3—C4—N1	−8.67 (18)	C16—C17—C18—C13	−1.7 (3)
N2—C3—C4—S1	1.5 (2)	C3—C4—N1—C13	−156.00 (14)
C2—C3—C4—S1	165.92 (11)	S1—C4—N1—C13	29.5 (2)
O3—C2—C6—O1	−40.0 (3)	C3—C4—N1—C1	−2.30 (16)
C3—C2—C6—O1	90.4 (2)	S1—C4—N1—C1	−176.76 (11)
C1—C2—C6—O1	−161.1 (2)	C18—C13—N1—C4	39.6 (2)
O3—C2—C6—C7	136.79 (15)	C14—C13—N1—C4	−143.72 (16)
C3—C2—C6—C7	−92.78 (16)	C18—C13—N1—C1	−112.28 (17)
C1—C2—C6—C7	15.68 (18)	C14—C13—N1—C1	64.36 (19)
O1—C6—C7—C8	164.5 (2)	O2—C1—N1—C4	136.14 (13)
C2—C6—C7—C8	−12.2 (2)	C8—C1—N1—C4	−100.46 (14)
O1—C6—C7—C12	−10.3 (4)	C2—C1—N1—C4	11.43 (15)
C2—C6—C7—C12	173.0 (2)	O2—C1—N1—C13	−68.05 (16)
C12—C7—C8—C9	1.3 (3)	C8—C1—N1—C13	55.35 (17)
C6—C7—C8—C9	−173.89 (16)	C2—C1—N1—C13	167.24 (13)
C12—C7—C8—C1	178.35 (18)	C4—C3—N2—O4	−23.3 (2)
C6—C7—C8—C1	3.1 (2)	C2—C3—N2—O4	173.83 (16)
O2—C1—C8—C7	−116.54 (15)	C4—C3—N2—O5	158.10 (16)
N1—C1—C8—C7	118.47 (14)	C2—C3—N2—O5	−4.8 (2)
C2—C1—C8—C7	6.84 (17)	N1—C4—S1—C5	28.50 (16)
O2—C1—C8—C9	60.2 (2)	C3—C4—S1—C5	−145.23 (14)

### Hydrogen-bond geometry (Å, º)

Cg1 is the centroid of the C13–C18 benzene ring.

**Table:**

*D*—H···*A*	*D*—H	H···*A*	*D*···*A*	*D*—H···*A*
O3—H3···O5	0.82	2.34	2.895 (2)	126
O2—H2···O5^i^	0.82	2.00	2.8155 (18)	171
C11—H11···O4^ii^	0.93	2.51	3.423 (3)	166
C9—H9···*Cg*1	0.93	2.89	3.545 (2)	129

Symmetry codes: (i) *x*+1/2, −*y*−1/2, −*z*; (ii) −*x*−1/2, −*y*, *z*+1/2.
